# Optimal Path of Internet of Things Service in Supply Chain Management Based on Machine Learning Algorithms

**DOI:** 10.1155/2022/4844993

**Published:** 2022-03-24

**Authors:** Jing Li, Ruifeng Zhang, Yanfeng Jin, Haiyan Zhang

**Affiliations:** Shijiazhuang Posts and Telecommunications Technical College, Shijiazhuang, Hebei 050021, China

## Abstract

With the development of global economic integration, the scale of supply chain network is becoming larger and larger. The research on the benefits of supply chain network under the Internet of Things technology is a new interdisciplinary research issue. Starting from the complexity and evolution of supply chain network, based on machine learning algorithms, this study analyses the service modes of the Internet of Things for supply chain enterprises, such as RFID technology, EDI technology, and enterprise resource planning ERP. Finally, by analyzing the degree of application of the Internet of Things in different industries, the manufacturing industry is selected for simulation. It is found that the introduction of the Internet of Things is conducive to speeding up the evolution of enterprise supply chain. Thus, the horizontal integration of the optimized path supply chain can effectively improve the overall profits of enterprises and provide theoretical support for the Internet of Things service supply chain.

## 1. Introduction

Today, with economic globalization, the supply and demand of the market are changing rapidly. Only by improving the ability of the supply chain to respond to the market quickly, enterprises can survive and develop [1]. In this environment, the smoothness, efficiency, and low cost of interenterprise logistics and information flow are particularly important.

Supply chain management (SCM) is a concept of design management science [2], which is based on information system technology and seamlessly integrates enterprise activities involved in the whole business process of supply chain. The efficient operation of supply chain is an important guarantee for the healthy development of enterprises [3]. At the same time, the management information of the managers is accurately received and implemented by the corresponding links of the supply chain, that is, the visualization and implementation management of the supply chain, which is the foundation of the efficient operation of the supply chain [4]. The Internet of Things and related technologies meet these needs of the supply chain.

The Internet of Things (IoT) aims at perceiving the physical world, takes information processing as its main task, and takes the network as its information exchange carrier to realize the information exchange between the object entity and the information management system [5]. In addition to the network composed of perceptual nodes, the Internet of Things can also fully connect with the Internet, mobile communication networks, and other networks. IoT is called the third wave of information industry development in the world after computer and the Internet through the integration and application of intelligent perception, identification technology, pervasive computing, and ubiquitous network [6].

## 2. Establishment of Supply Chain Management Model

### 2.1. Supply Chain Management Concept

Supply chain management (SCM) is the whole process of putting raw materials into the market through the division of labor and cooperation of enterprises in different links of product design, production, and transportation on the premise of satisfying consumers' needs. Supply chain management combines consumers, products, and channel supply organically [7] and realizes close cooperation among enterprises with different divisions of labor in the supply chain system. It is the whole process of products from demand to design to meet consumers' needs in kind.

The main objects of supply chain management are divided into three aspects: capital flow, logistics flow, and information flow in the supply chain system. The process of optimizing the supply chain operation process through technology to achieve efficient operation of the supply chain system, starting from procurement to the consumer, which provides the right product at a reasonable price is supply chain management [8]. Supply chain management involves all aspects of supply chain operation, including raw material suppliers, manufacturers, distributors, retailers, and consumers. Generally speaking, the supply chain is aimed at manufacturing enterprises, that is, manufacturers. The upstream enterprises are called raw material suppliers, while the downstream enterprises are called raw material distributors, retailers, and so on. In different supply chain systems, the role of enterprises is relative. For example, a component manufacturer is presented as a manufacturer in its supply chain system, and its component manufacturing raw material enterprise is presented as a supplier. In another supply chain system, the component manufacturer may act as its downstream enterprise. For example, in the supply chain system of automobile and furniture manufacturers, the component manufacturer may act as its upstream enterprise and present as its supplier [9]. The complete supply chain function system includes all steps of supply chain management, such as planning, obtaining, storing, distributing, and serving.

Supply chain management has the characteristics of market response and organization scale. It is an important development system accompanied by consumption and production. It provides a complete product circulation process from manufacturer to market and has a direct effect on the final marketing activities of products.

### 2.2. Supply Chain Management Process

With the continuous change of market environment and the continuous improvement of production technology, the demand of users presents a diversified trend. The product structure is becoming more and more complex. For the member enterprises in the supply chain, the market demand is becoming more and more difficult to grasp. For supply chain managers, the competitiveness of member enterprises in the supply chain must be improved by strengthening the composition and operation management of the supply chain. Under the influence of a highly competitive internal and external environment, an efficient and stable supply chain has become the goal of many enterprises. The supply chain network with good stability and adaptability is gradually replacing a single supply chain.

**Figure 1:**
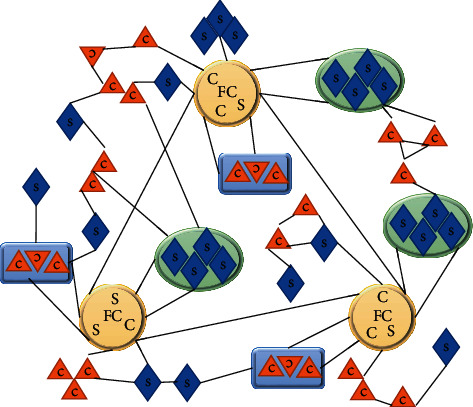
Schematic diagram of supply chain network structure. S, supplier; FC, core enterprise; C, customer.

In mathematical graph theory, there are many different descriptions of networks [10]. In mathematical language, a network is a graph that represents the objects of study and their relations with each other. The physical meaning of network is a model abstracted from similar problems and expressed and studied by graph theory. A network is a graph composed of many vertices (nodes) and edges connecting these vertices, in which vertices represent different entities in a real system and edges represent the relationships among these entities. If there is a specific relationship between the two vertices, then there is an edge between the two vertices, and the two vertices are called neighbors to each other. If there is no relationship between the two vertices, there is no edge connection between the two vertices, as shown in Figure 1.

The process of supply chain management is based on the strategic objectives of different types of production enterprises. It is a collection of various means realized by using supply chain technology in product planning, design and development [11], production and sales, etc. In general, the supply chain management process includes the following steps:

1. Plan. As one of the five functions of management theory, planning is the starting point for a series of activities such as product planning and design. Supply chain management planning refers to all the design and planning processes of product supply according to enterprise market conditions and strategic planning, comparing the process of terminal product delivery to customers. Consumer and market forecasting are prerequisites. The plan is made after defining the internal and external conditions. Plans in supply chain management include product markets, consumer demand, product development, supply, replenishment, etc. The demand information transmission in the supply chain can be directly from retailers to distributors, or from retailers to manufacturing enterprises, or even to raw material suppliers, finished products logistics, and so on.
2. Implementation. The practical problems often encountered in supply chain management make the larger productivity and smaller inventory volume unable to match. The method effectively reduces production costs, meets customer needs to find a balance, and maximizes the rational use of human and material resources to improve supply chain efficiency. The implementation of supply chain management activities is a process based on the integration of supply chain resources allocation, aiming at optimizing the efficiency of supply chain management. It will include product research, raw material procurement, and manufacturing and distribution services as members of the cooperative operation system.
3. Assessment. The process of supply chain evaluation is to track the product itself, supply chain operation, capital operation, and other aspects [12]. Through the evaluation results, open policy and system construction are carried out, customers and markets are effectively responded to, and new supply chain management technology is applied. For example, radio frequency identification (RFID) and Electronic Label technology are used to realize the quality control, information transmission, and production plan execution in the process of supply chain management. One of the key problems is the quantitative evaluation of financial management system, which is an enterprise of supply chain management system. Because of its operation status, it can measure and control the system.

### 2.3. Supply Chain Evolution Model

**Figure 2:**
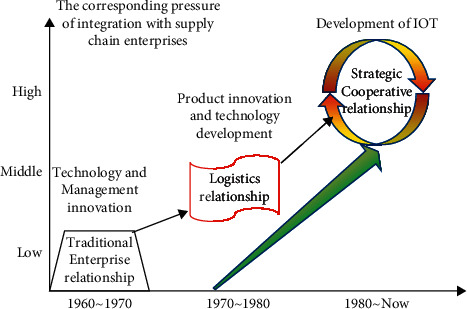
The development of cooperation between enterprises in supply chain.

In the research of supply chain management, there are many problems about cooperation among enterprises. Most researchers believe that the key to the success of supply chain management is to properly handle the cooperation among enterprises in the supply chain [12]. The development of enterprise relationship in supply chain management has gone through three stages: traditional enterprise relationship, logistics relationship, and strategic cooperative relationship. The specific process is shown in Figure 2.

**Figure 3:**
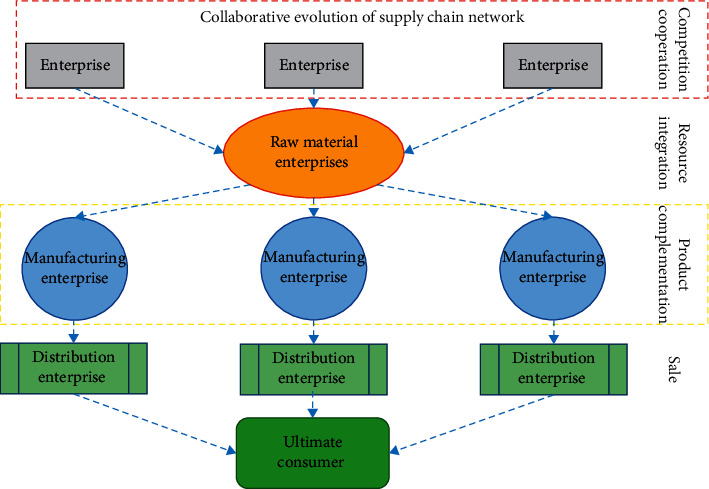
Synergistic evolution of enterprises in supply chain networks under hierarchical structure.

In this study, each vendor in the supply chain network is regarded as a “node” in the complex network, and the various cooperative relationships among vendors are regarded as “edges” in the complex network. Through information flow, logistics, and capital flow, firms can achieve cooperative relations with each other, thus forming a complex supply chain network [13]. In order to better understand the evolution mechanism of supply chain network, this study analyses the entry, exit, and cooperation behaviors of vendors in the supply chain network from the perspective of the cooperative relationship shown by node vendors, as shown in Figure 3.

The BA evolutionary model follows two basic evolutionary mechanisms: “growth” and “preferential connection.” Since BA model was put forward, scholars began to use BA model extensively to conduct in-depth research on the supply chain network. Based on the BA model, considering the entry, exit, and cooperation behaviors of nodes, this study extracts the evolution rules of supply chain network according to the evolution characteristics of supply chain network, as listed in Table 1.

In the process of manufacturer's gradual withdrawal from the supply chain network, that is, when the connection in the network is gradually disconnected, this study assumes that in each time step, the *n* connection is disconnected with the antioptimal probability. Within the supply chain network, new cooperative links are always established among different manufacturers, which also follow the selection. Based on the above analysis, this study proposes a machine learning algorithm for supply chain network evolution that integrates the entry, exit, and cooperation mechanisms of manufacturers [14]. The specific algorithm is as follows:

Starting from *m*_0_ isolated node vendors, and in each time step, the following three situations occur:

(1) Adding a new node manufacturer with *m*(*m* ≤ *m*_0_) edge, two endpoints of each new edge are connected to the existing node *i* by probability ∏_*i*_, where *k*_*i*_ is the degree of node *i* and ∑_*j*_*k*_*j*_ is the sum of node degrees.
  (1) $$\begin{array}{c} \prod_{i}=\frac{k_{i}}{\sum_{j}k_{j}}. \end{array}$$
(2) Disconnection. Disconnecting *n* edge from the network, both ends of the disconnected edge are selected with the antipreferential probability ∏_*i*_′. The probability that the old node *i* becomes one end of the disconnected edge is as follows:
  (2) $$\begin{array}{c} \prod_{i}^{\prime }=\frac{1-\prod_{i}}{N\left(t\right)-1}. \end{array}$$
   Here, *N*(*t*) is the number of nodes in the network, that is, *N*(*t*)=*t*+*m*_0_, *m*_0_ is the initial manufacturing quantity, and *t* is the number of cycles.
(3) Cooperative connection. *r* new edge is added to the network, and the cooperation between these edges and node *i* also follows the preferential connection, that is, the two endpoints of the new edge are selected with the preferential probability *i*.

These three situations explain the process of firms' entry, exit, and cooperation in supply chain network. With the evolution of time, under the influence of these three mechanisms, supply chain network has gradually developed into a complex network.

We assume that the comprehensive benefit growth of core enterprise *x*_*i*_ among nonreciprocal enterprises obeys the growth law, that is, d*x*_*i*_(*t*)/d*t*=*γ*_*i*_*x*_*i*_(1 − *x*_*i*_/*N*_*i*_). For member enterprise *x*_*j*_, if there is no core enterprise *x*_*i*_, the business of member enterprise will probably shrink in the market competition and then withdraw from the supply chain network. Therefore, the development law of member enterprise *x*_*j*_ can be described as d*x*_*j*_(*t*)/d*t*=−*r*_*j*_*x*_*j*_.

The evolution model of nonreciprocal cooperative relationship between core enterprises and member enterprises in supply chain network can be described as follows:

(3) $$\begin{array}{c} \left\{\begin{array}{c} \frac{\text{d}x_{i}\left(t\right)}{\text{d}t}=\gamma_{i}x_{i}\left(1-\frac{x_{i}}{N_{i}}+\delta_{ij}\frac{x_{j}}{N_{j}}-\sigma_{ij}\frac{x_{j}}{N_{j}}\right),\\ \frac{\text{d}x_{j}\left(t\right)}{\text{d}t}=r_{j}x_{j}\left(-1-\frac{x_{j}}{N_{j}}+\delta_{ji}\frac{x_{i}}{N_{i}}-\delta_{ji}\frac{x_{i}}{N_{i}}\right). \end{array}\right) \end{array}$$

The process of solving the equilibrium point is as follows:

(4) $$\begin{array}{c} \left\{\begin{array}{c} f\left(x_{i},x_{j}\right)=\frac{\text{d}x_{i}\left(t\right)}{\text{d}t}=\gamma_{i}x_{i}\left(1-\frac{x_{i}}{N_{i}}+\delta_{ij}\frac{x_{j}}{N_{j}}-\sigma_{ij}\frac{x_{j}}{N_{j}}\right)=0,\\ g\left(x_{i},x_{j}\right)=\frac{\text{d}x_{j}\left(t\right)}{\text{d}t}=r_{j}x_{j}\left(-1-\frac{x_{j}}{N_{j}}+\delta_{ji}\frac{x_{i}}{N_{i}}-\delta_{ji}\frac{x_{i}}{N_{i}}\right)=0. \end{array}\right) \end{array}$$

Only for nonreciprocal cooperative enterprises, when the influence of core enterprises on member enterprises is greater than that of member enterprises on core enterprises, the cooperative competition among nonreciprocal enterprises can reach a stable equilibrium state. This is consistent with the reality of supply chain management. The enterprise that plays a key leading role in promoting the business operation of the whole supply chain, which can not only provide customers with maximum added value but also help other cooperative enterprises in the chain to participate in the new market, is the core enterprise of the supply chain, also known as the leader enterprise of the supply chain. Other enterprises in a relatively secondary position are called noncore enterprises of supply chain management. In the satellite supply chain, the only main enterprise is the core enterprise of the supply chain. In the team supply chain, the core enterprise is also the only one, but it is not fixed. It changes dynamically with the changes of the main business of the supply chain, the transfer of scarce resources, the evolution of the market environment, and other factors. Next, we explain the change of the equilibrium point of the model by analyzing the phase plane trajectory of equation (4).

**Figure 4:**
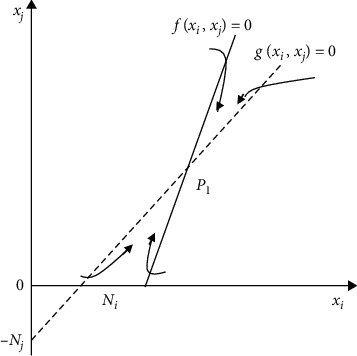
Stability plane trajectory of equilibrium point in enterprise cooperation and competition.

As shown in Figure 4, the phase plane of equation (4) is divided into four regions and the trajectory will eventually tend to equilibrium point *P*_1_ regardless of any point in the region from which it starts. This shows that the nonpeer enterprises in the supply chain network will eventually reach a stable cooperative state under the relationship of cooperation and competition.

## 3. Supply Chain-Oriented Internet of Things Technical Service Characteristic System

The Internet of Things (IoT), also known as “sensor network,” refers to the use of radio frequency identification (RFID) and other information sensing technology. Intelligent management and identification can be achieved by linking the information of all items (including materials/spare parts/in-process/finished products, etc.) in the supply chain with the Internet in real time.

### 3.1. Basic Characteristics of Internet of Things

The Internet of Things (IoT) consists of three layers: firstly, the sensing layer realizes the identification of things, that is, identifies “things” with various sensing technologies. Among them, RFID technology is the main representative [15]. Secondly, the transmission layer realizes the transmission and sharing of information, that is, through the existing LAN, WAN, Internet, communication network, as well as EPC, EDI, and other data analysis and exchange technologies, to achieve data transmission. Thirdly, the application layer realizes the processing and application of the acquired perceptual data information. It includes application program and display terminal. The application program is installed on the operating system of mobile phone, computer, and other mobile terminals and is applied according to business logic.

“Perception” is the first step in the implementation of the Internet of Things. The perception layer is used to recognize, collect, and capture the internal attributes (intrinsic characteristics of the object itself) and external attributes (environmental characteristics of the object), that is, the “feeling” in “perception.” On this basis, the collected data are preprocessed with preliminary judgments and filters, that is, the “knowledge” in “perception”. “The sensing layer mainly includes RFID technology, GPS technology, and other sensing technologies. In the supply chain, the material perception function is mainly realized through RFID technology, GPS technology, and so on.

The transport layer needs to integrate all kinds of network transmission, as well as the analysis of related servers, to enhance the data acquired by the perception layer to the level that can be used by the application layer [16]. The transport layer is the infrastructure of the Internet of Things. It needs to play the role of the perception layer and the application layer.

In the Internet of Things, which is composed of an electronic tag, reader, middleware (including electronic code information matching server), and information data application system, the reader transmits the read electronic code to the electronic code information matching server after preliminary data processing by the underlying middleware. The article details corresponding to the electronic coding are matched with the electronic coding, and the matched details are transmitted to the application system for business logic application. In the process of transportation, the vehicle can send the position information obtained by the GPS back to the GIS system through the GSM/GPRS network for application.

### 3.2. Core Technology of Internet of Things

#### 3.2.1. Radio Frequency Identification (RFID) Technology

RFID is the abbreviation of radio frequency identification, also known as electronic tags. RFID technology is a noncontact automatic identification technology based on the principle of radio frequency. It automatically identifies target objects and acquires relevant data through radio frequency signals. Different from other identification technologies, RFID technology has low environmental requirements and high sensitivity to electronic tags. RFID technology can identify the static or high-speed moving targets efficiently and automatically [17]. It can also identify tags in batches at the same time. It can effectively solve the multiobjective batch reading business, which often occurs in supply chain management. It ensures an accurate and convenient acquisition of target information data.

#### 3.2.2. EDI Technology

Electronic data interchange (EDI) is a kind of data exchange technology. The specific realization in supply chain management refers to the unified standards accepted by both sides between enterprises that have economic exchanges. Data, documents, and reports related to business transactions are transmitted and processed by computer through the network.

#### 3.2.3. Enterprise Resource Planning (ERP)

ERP system is the basis of enterprise supply chain management based on Internet of Things technology and also can bring the potential of intelligent supply chain into full play. For enterprises, using Internet of Things technology, the object of ERP system can be extended to goods, and lean management can be carried out by more specific physical entities [18], so as to optimize the flow of resources within enterprises and improve production efficiency, ultimately achieving efficient and high-quality external services. Essentially, the ERP system is the means to realize the information of supply chain management. It does not change the content and mode of supply chain management but only speeds up the information transmission speed of supply chain management.

#### 3.2.4. Geographic Information System (GIS)

With the support of computer hardware and software systems, GIS is a technical system for inputting, storing, and analyzing spatial geographic data. Its most prominent features lie in two aspects. On the one hand, it can show the data in the form of electronic map, so that the data can be seen, touched, image, and vivid. On the other hand, it can perform spatial analysis and computation.

Using GIS technology in distribution management, customer location can be marked on electronic map and distribution route can be optimized based on method model. The application of GIS technology in transportation management can monitor the transportation vehicles equipped with GPS in real time, plan the transportation lines, and visualize the whole transportation process.

## 4. Optimizing Path of Supply Chain Management System under the Background of Internet of Things

### 4.1. Vertical Integration Path

**Figure 5:**
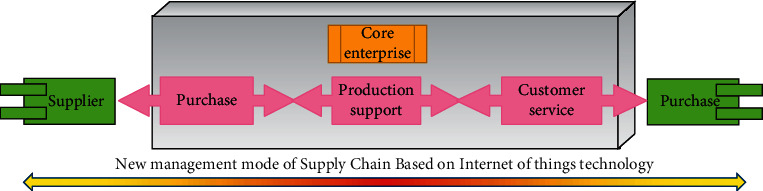
Vertical integration path of supply chain management under the Internet of things technology.

Vertical integration path refers to the process of diffusion and promotion of the new supply chain management model based on Internet of Things technology along the core enterprises of the supply chain to the downstream enterprises. As the Internet of Things is still a new information technology, the new supply chain management model based on the Internet of Things technology is still in the exploratory stage [19]. The application mode and application value of Internet of Things technology in the supply chain are still unclear. The application of Internet of Things technology is complex and the cost of investment is high. And its application in the supply chain involves the complex cooperative relationship among the members of the supply chain. There is a serious “free-rider” problem between the members of the supply chain, which hinders the rapid adoption and promotion of Internet of Things technology in the supply chain. As the core enterprise in the supply chain, it can often get the most benefit from the adoption of Internet of Things technology. Therefore, its motivation to adopt Internet of Things technology is the strongest. On the one hand, it has strong technical capability and abundant resources to be the first to introduce IoT technology into the enterprise. On the other hand, it can take advantage of the core position of upstream and downstream enterprises in the supply chain and exert pressure on them to adopt IoT technologies to meet the core enterprises' own development needs. In this way, a new supply chain management model based on Internet of Things technology has gradually formed and gradually spread to the whole supply chain along the upstream and downstream of the supply chain. The vertical integration path to the new supply chain management mode based on Internet of Things technology is shown in Figure 5.

**Figure 6:**
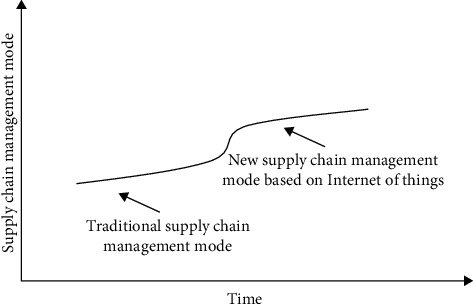
Development mode of vertical integration new supply chain management model.

According to the functions and characteristics of the Internet of Things technology, the original supply chain management system is constantly expanded, so that the original supply chain management mode gradually transits to a new supply chain management mode based on the Internet of Things. This path is suitable for the supply chain management system, which has already been mature, and the existing management mode is relatively stable. The supply chain with seamless embedding of Internet of Things technology is mostly in its mature stage and has a relatively stable management mode. The fierce competition and the urgency to adopt the Internet of Things technology are not great [20], but in order to maintain the competitiveness of the supply chain, we still need to adopt the Internet of Things technology to improve competitiveness. In order to avoid the violent fluctuation of supply chain management, this kind of supply chain tends to adopt the gradual path to gradually adopt the Internet of Things technology and the adjustment of management mode. The gradual path to a new supply chain management model based on Internet of Things is shown in Figure 6.

### 4.2. Horizontal Integration Path

**Figure 7:**
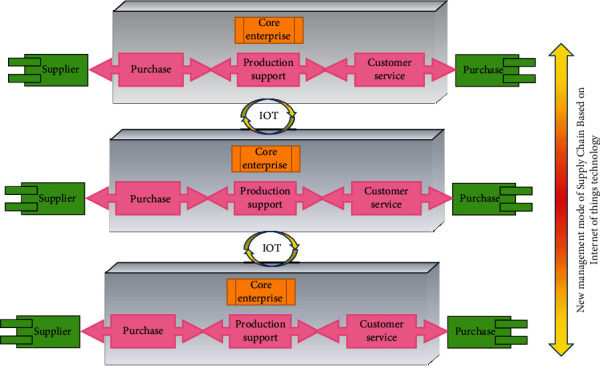
Vertical integration path of supply chain management under Internet of things.

Horizontal integration path refers to the process of diffusion and promotion of a new supply chain management model based on Internet of Things technology from the supply chain that formed the model earlier to the supply chain in the industry or other industries. In the process of adopting Internet of Things technology, due to the different characteristics and situations of different industries and supply chains, the maturity of Internet of Things technology used by them also varies. Industries with high visibility requirements such as food, retail, logistics, and some subindustries in the manufacturing industry may take the lead in adopting Internet of Things technology to explore its application value and management mode. For other industries that do not require high visibility of information, the application value of Internet of Things technology will take longer or wait until other industries have formed a more mature model before it emerges [21]. In addition, due to the constraints of technology, scale, and strength of enterprises in different supply chains in the same industry, there are obvious differences in their exploration and adoption of Internet of Things technology. Industry leaders and some enterprises with high technology sensitivity may take the lead in exploring the use of Internet of Things technology to test the change of management mode. For those supply chains with weak technological strength and small scale, the risk of taking the lead in exploring Internet of Things technology is high. Therefore, they are more inclined to imitate the mature model of industry leaders or forced to follow the competitors to adopt Internet of Things technology and change the management model under the pressure of competition. This constitutes a horizontal integration path to a new supply chain management model based on Internet of Things technology, as shown in Figure 7.

The supply chain is in the initial or recession period of supply chain growth, either it has not yet formed a supply chain management system or its original supply chain management system has been seriously outdated and aging. In this case, the cost of directly using the new generation of information technology of the Internet of Things in the supply chain is much less than that of gradual adoption of the Internet of Things technology and has a high necessity. Therefore, for the supply chain in the above situation, it tends to adopt a leapfrog path to achieve supply chain management based on Internet of Things technology and form a new management model on this basis to meet the needs of competition.

The new supply chain management mode based on the Internet of Things technology is designed, and the design principles and ideas of the new supply chain management mode based on the Internet of Things technology are given. On this basis, the function and management changes of the new supply chain management mode based on the Internet of Things technology are analyzed. Compared with the traditional supply chain management mode [22], the adoption of Internet of Things technology can increase the transparency and visibility of the supply chain. The uncertainty in the supply chain is eliminated and the level of supply chain intelligence and other new functions are improved. Correspondingly, the supply chain management mode has changed its management function and management links.

## 5. Simulation Results

**Figure 8:**
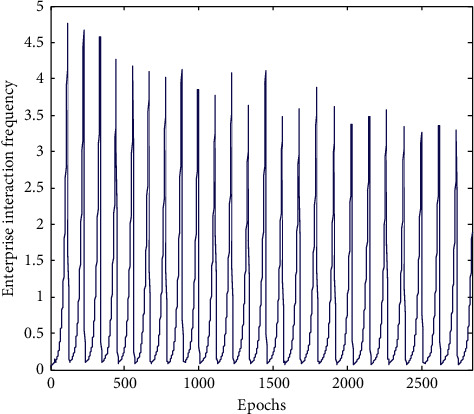
Simulation diagram of enterprise evolution frequency.

**Figure 9:**
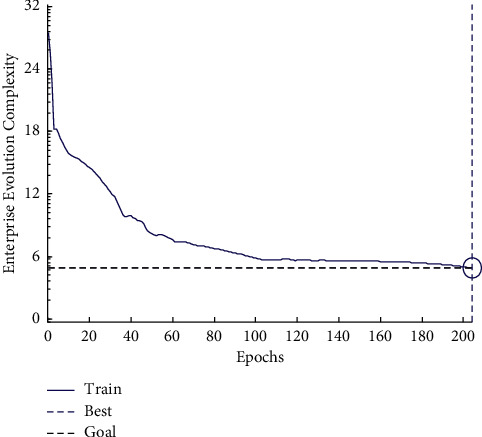
Enterprise evolution complexity map.

The main industries using Internet of Things technology are manufacturing, logistics, warehousing, and postal industry, while the proportion of wholesale and retail industries using Internet of Things technology is relatively small. It shows that the vast majority of wholesale and retail enterprises in China have not yet realized the great role of technology in improving their operations or the current implementation is still facing greater difficulties. From the perspective of manufacturing subindustries, the industries with more technology are communication and related equipment manufacturing and computer and related equipment manufacturing. These industries are highly related to information technology [23]. This study chooses the related industries of manufacturing and sales industry in a certain area, including e-commerce sales and offline sales enterprises. The Internet of Things database is designed on the basis of the original ERP database. The data table includes the order form and order detail table related to the order. Material discharging table, main production plan table, detailed production plan table, production plan list, process route table, and work center table related to production. Material warehousing information table, finished product warehousing table, and finished product outgoing table related to warehousing, and basic vehicle information table and vehicle scheduling table related to distribution. Vehicle operation information table, vehicle bill of materials table, and vehicle bill of materials detailed table related to transportation. By simulating the operation process of the whole sales network, the reasonable number of enterprises participating in the network is obtained. The frequency of enterprise evolution and the complexity of enterprise evolution are respectively shown in Figures 8 and 9.

The analysis chart and the conclusion are as follows: under the optimal replacement rule, the number of enterprises changes alternately, the degree of cooperation between node enterprises decreases slightly with the increase of iteration steps, and the change of parameters reaches a stable state. Finally, it shows that the number of sales enterprises is about 6 to reach the optimal state.

Four sets of numerical experiments were conducted to keep the parameters of the first product the same as those of the second product, while changing the profit margin, average demand, inventory loss, and product substitution ratio of the second product to make them different from the relevant parameters of the product. The main object of our study is the optimal order quantity of two products, their corresponding expected profits, and their corresponding sums, that is, the impact of integrating inventory information and adopting Internet of Things technology on product order quantity and product sales, respectively. In addition, it compares the degree of optimization of product order quantity without using the Internet of Things to optimize the industrial chain.

**Figure 10:**
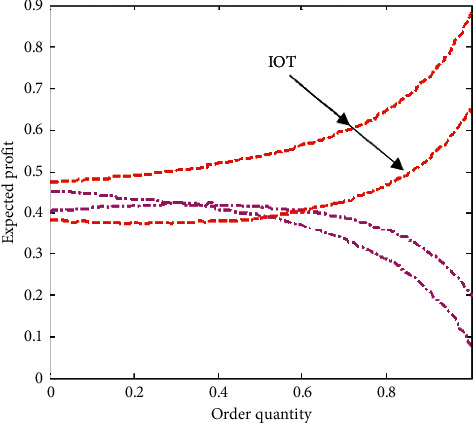
Contrast of different technologies impact on expected profit.

**Figure 11:**
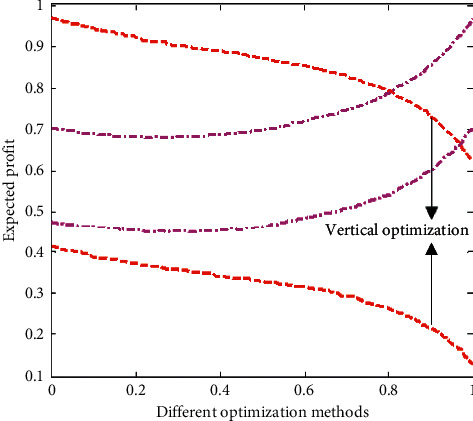
Contrast of the effects of different optimizations on expected profit.

As shown in Figure 10, under the Internet of Things technology, the expected profit of products has an inverted U-shaped relationship with the number of orders, while under the traditional information integration, the overall profit of products decreases significantly with the increase of the number of orders, because the inefficiency of information transmission increases the operating cost of enterprises. As shown in Figure 11, compared with the vertical integration optimization, the horizontal integration optimization of supply chain has obvious advantages, which shows that the communication and cooperation between enterprises in different industrial chains accelerate the exchange frequency of goods and improve the efficiency of enterprise operation.

## 6. Conclusion

The supply chain is a functional network chain structure that connects suppliers, manufacturers, distributors, retailers, and end users into a whole by controlling information flow, logistics, and capital flow, starting from purchasing raw materials, making intermediate products and final products, and finally sending products to consumers by the sales network. It is not only a logistics chain, information chain, and capital chain connecting suppliers to users but also a value-added chain. Materials increase their value in the supply chain due to processing, packaging, transportation, and other processes, bringing benefits to relevant enterprises. In the scale-free cooperation of supply chain network enterprises, a higher initial degree of cooperation will lead to a long-term stable cooperation state in the process of enterprise evolution, forming a complex common network of enterprises. Starting from the application of the Internet of Things technology in the manufacturing industry, this study analyses the technical support of the Internet of Things technology in the process of enterprise operation and carries out a simulation to draw the following conclusions:

In the evolution process of complex networks, the number of enterprises changes alternately and the degree of cooperation between node enterprises decreases slightly with the increase of iteration steps. Finally, the number of complex network nodes that maximize profits in the region is obtained. The introduction of Internet of Things technology is conducive to accelerating the evolution of enterprise supply chain.

Under the Internet of Things technology, the horizontal integration optimization of supply chain has obvious advantages, improves the efficiency of enterprise operation, and provides a theoretical basis for the optimization of supply chain.

## Figures and Tables

**Table 1:** Supply chain evolutionary network rules.

Rulemaking	Rule description
Growth rule	Supply chain network effectively integrates dispersed resources and technologies, has corresponding economic effects and cluster advantages, and will attract a large number of manufacturers to join the network.
Preferred connection rules	When choosing partners, new suppliers who enter the supply chain network follow the rules of preferential connection. New suppliers are willing to choose the core suppliers who have more cooperative relationships in the supply chain network to cooperate.
Cooperation rules	In the supply chain network, there are always new cooperative links between manufacturers and other manufacturers and various links occur.
Exit rule	Due to the fierce competition among manufacturers in the supply chain network, some firms will have greater pressure to survive and will choose or be forced to terminate their cooperative connections with each other.
Anti-preferential is connection rule	The greater the degree of node, the less likely the connection not has to be lost.

## Data Availability

The data used to support the findings of this study are available from the corresponding author upon request.
